# 382. Are errors of omission falsely inflating hospital-onset Clostridioides difficile infection numbers?

**DOI:** 10.1093/ofid/ofac492.460

**Published:** 2022-12-15

**Authors:** Jorge Paiva Parada, Mohamed Yussif, Louise Lie

**Affiliations:** Loyola University Chicago, La Grange, Illinois; Loyola University Medical Center, Maywood, Illinois; Loyola University Medical Center, Maywood, Illinois

## Abstract

**Background:**

Identification of *C. difficile* infection (CDI) as community onset (CO) versus hospital onset (HO) is based upon the timing of the laboratory testing. Any lab diagnosis made after day 3 of hospitalization is classified as HO-CDI, even if there is clinical evidence that the patient had CDI before lab testing was sent on day 4 or later. Capturing CDI infection early in hospitalization is essential to proper diagnosis and management of CDI.

**Methods:**

This is a single center retrospective study of all HO-CDI cases identified from January 2018 through January 2022. All cases were reviewed to determine if patients had loose stools during days 1-3 which were not tested, as these may have been missed opportunities to diagnose CO-CDI. We used nursing flowsheet determine if the patient had soft, loose, watery, liquid or pasty stool bowel movements (BM).

**Results:**

We identified 302 unique patients diagnosed with HO-CDI during the 4 year study period. 181 (60%) were men. The mean age 57 (range 3-98 years), with increasing case numbers with increasing age groups: age 0-18 (19/6.3%), 19-39 (39/12.9%), 40-59 (73/24.2%), ≥ 60 (171/56.6%). Mean time of HO-CDI diagnosis was 12.4 days (range 4-122 days) with a skew towards early onset HO-CDI diagnosis as 86/302 (28.5%) testing positive on days 4-6. Overall, 119/302 (39.0%) of patients had identified missed opportunities for testing stools during days 1-3 of hospitalization. 38/86 (44.2%) of patients with HO-CDI diagnosed on days 4-6 had missed opportunities for testing. 40/302 (13.2%) patients expired during the index hospitalization.

**Conclusion:**

Better tracking of patients' symptoms, number and consistency of BMs could improve recognition of patients presenting with CO-CDI. In turn, this would help to institute earlier isolation and treatment of these patients, as well as decreasing HO-CDI resulting from late recognition of CO-CDI which may falsely inflate HO-CDI case numbers, and/or lead to secondary hospital transmission from delayed isolation of patients with CDI. Our findings likely represent a significant under estimation of the magnitude of the errors of omission of missing opportunities for early diagnosis of CDI since it based on review of nursing flowsheets which often miss the number and consistency of patients' BMs.

**Disclosures:**

**Jorge Paiva Parada, MD MPH**, Shionogi: Honoraria.

